# Stability of SiN_X_/SiN_X _double stack antireflection coating for single crystalline silicon solar cells

**DOI:** 10.1186/1556-276X-7-50

**Published:** 2012-01-05

**Authors:** Youngseok Lee, Daeyeong Gong, Nagarajan Balaji, Youn-Jung Lee, Junsin Yi

**Affiliations:** 1 Department of Energy Science, Sungkyunkwan University, 300 Chunchun-dong, Jangan-gu, Suwon, 440-746, South Korea; 2 School of Information and Communication Engineering, Sungkyunkwan University, 300 Chunchun-dong, Jangan-gu, Suwon, 440-746, South Korea

**Keywords:** SiN_X_, PECVD, double stack, stability, temperature, humidity test.

## Abstract

Double stack antireflection coatings have significant advantages over single-layer antireflection coatings due to their broad-range coverage of the solar spectrum. A solar cell with 60-nm/20-nm SiN_X_:H double stack coatings has 17.8% efficiency, while that with a 80-nm SiN_X_:H single coating has 17.2% efficiency. The improvement of the efficiency is due to the effect of better passivation and better antireflection of the double stack antireflection coating. It is important that SiN_X_:H films have strong resistance against stress factors since they are used as antireflective coating for solar cells. However, the tolerance of SiN_X_:H films to external stresses has never been studied. In this paper, the stability of SiN_X_:H films prepared by a plasma-enhanced chemical vapor deposition system is studied. The stability tests are conducted using various forms of stress, such as prolonged thermal cycle, humidity, and UV exposure. The heat and damp test was conducted for 100 h, maintaining humidity at 85% and applying thermal cycles of rapidly changing temperatures from -20°C to 85°C over 5 h. UV exposure was conducted for 50 h using a 180-W UV lamp. This confirmed that the double stack antireflection coating is stable against external stress.

## Background

Silicon nitride films are widely used in semiconductor device industries as well as in photovoltaic industries due to their strong durability, good dielectric characteristics, and resistance against corrosion by water [1,2]. Hydrogenated silicon nitride films can improve reflectance and surface passivation [3].

A single-layer antireflection coating is known to be unable to cover a broad range of the solar spectrum [4,5], and using double-layer antireflection coating is considered. There have been reports of using double-layer antireflection coatings of two different materials, such as MgF_2_/CeO_2_, SiO_2_/TiO_2_, MgF_2_/TiO_2_, SiO_2_/SiN, and MgF_2_/ZnS [6-8]. Two materials with different refractive indices are stacked together for double stack antireflection coating. This may be more vulnerable to outside stress. Solar cells operate in an external environment, and it is important that the surface of the solar cells endures various kinds of physical conditions. Thus, the antireflection film of solar cells should have strong resistance against a number of stress factors. SiN_X_:H thin film is often used as antireflection coatings. Its stability against ultraviolet light should be verified since it absorbs most of the ultraviolet light of the short wavelength region [9]. SiN_X_:H thin films deposited by plasma-enhanced chemical vapor deposition [PECVD] contain about 8% to approximately 30% (atom) hydrogen and are easily affected by moisture. Thus, the analysis of the stability against various stresses is necessary. However, little research has been conducted on the stability of SiNx used as antireflection coating [ARC] or solar cells.

In this paper, the stabilities of SiN_X_:H thin films deposited under various conditions and double stack SiN_X_:H thin films with different refraction indices are studied by applying different kinds of stress. Solar cells with double stack antireflection coatings are fabricated, and their characteristics are analyzed.

## Methods

Single layers of SiN_X_:H thin films are first studied to find the appropriate deposition conditions and to verify the stability and reliability of the double stack antireflection coating. A p-type crystalline silicon wafer with a sheet resistance of 1 to approximately 3 Ω cm and < 100 > orientation is used as the substrate for the deposition of thin films. The wafer is doped with phosphorous in a furnace using a conventional POCl_3 _diffusion source at 830°C for 7 min. Phosphorus silicate glass [PSG] is removed by dipping the wafer in 10% hydrofluoric acid [HF] solution for 30 s. The drive-in process is conducted for 25 min at 860°C. Next, a second doping process at 810°C is followed for 7 min. PSG is removed by dipping the wafer in 10% HF solution for 30 s. SiNx deposition is conducted in the environment of N_2 _at 450°C with a radio frequency [RF] power of 180 mW/cm^2^. The ratio of SiH_4_:NH_3 _is varied. The flow rate of NH_3 _is fixed at 200 sccm, and the flow rate of SiH_4 _is varied. Double stack SiN_X_:H with refractive indices ranging from 1.9 to 2.3 is prepared. All the samples are co-fired in a conveyer belt furnace. The effective minority carrier lifetimes are determined with the microwave photo conductance decay technique via quasi-steady state photoconductance using the WCT-120 silicon wafer lifetime detector (Sinton Consulting Inc., Boulder, CO, USA) before and after applying a stress. Fourier transform infrared spectroscopy [FT-IR] characteristics are measured using Shimadzu IR Prestige-21 (Shimadzu Corporation, Nakagyo-ku, Kyoto, Japan).

A temperature cycle with a maximum of 80°C and a minimum of -20°C within 5 h is used to test the stability against the temperature of SiN_X_:H thin films. Twenty temperature cycles, i.e., 100 h, are applied to see the effect of the constantly changing temperature, fixing the humidity at 85%.

The samples are exposed to ultraviolet light using a 180-W UV lamp to test the stability against ultraviolet rays. First, they are exposed to the UV light for 5 min six times; 15 min for the next six times; 30 min for the next six times; 1 h, five times; and then 10 h, five times.

Finally, the solar cells are fabricated. A 5-in. p-type crystalline CZ-Si solar-grade wafer of around 200 μm thick having a specific resistance of around 1 to approximately 3 Ω cm with < 100 > orientation is used as the substrate for the deposition of thin films. A 2% NaOH solution is used for pyramidal texturing of the Si wafer. The wafers are dipped for 25 min in the 2% NaOH etching solution maintained at 84°C to approximately 86°C. All textured p-type silicon wafers are then doped with phosphorus in a furnace using a conventional POCl_3 _diffusion source first at 830°C for 7 min. PSG is removed by dipping the wafer in 10% HF solution for 30 s. Then, the drive-in process is conducted at 860°C for 25 min. The second doping is done at 810°C for 7 min. The SiN_X _film is then deposited on the substrate using the PECVD technique. During deposition, RF power, plasma frequency, pressure, and substrate temperature are maintained at 180 mW/cm^2^, 13.56 MHz, 0.5 to approximately 0.8 Torr, and 450°C, respectively. The gas flow rates of NH_3 _and N_2 _are maintained at 200 sccm and at 85 sccm, respectively, for the double stack antireflection coating of the SiN_X_:H film on the silicon wafer; whereas, the SiH_4 _flow rate is set at 20 and 80 sccm for each layer. Back metallization is conducted with a standard aluminum paste using the screen-printing technique. The samples are then baked and co-fired in a conveyer belt furnace. The effective carrier lifetime and efficiency characteristics are measured using Sinton WCT-120 (Sinton Consulting Inc., Boulder, CO, USA) and Pasan cell tester CT 801 (Pasan Measurement Systems, Neuchâtel, Switzerland). Reflectance characteristics are measured using Scinco S-310 (Scinco S-310, Seoul, Korea).

## Results and discussion

**Figure 1:**
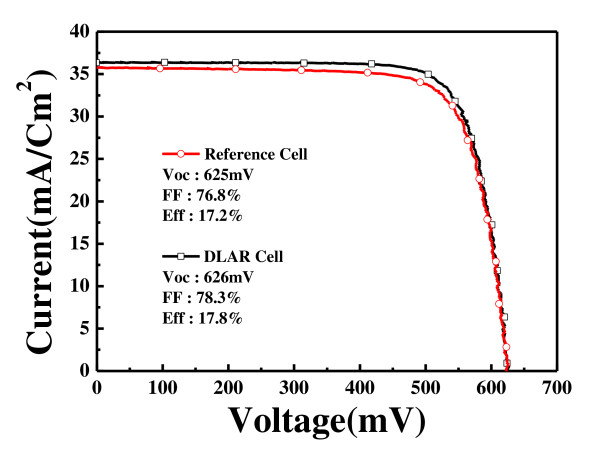
***I*-*V *characteristics of solar cells with double stack antireflection coatings of SiN_X_/SiN_X_**. The reference cell only has a single-layer SiN_X_.

The solar cells with double stack antireflection coating have better cell characteristics than those with single-layer antireflection coating. The double stack antireflection coating proves to have a better passivation effect than the 80-nm-thick single-layer SiN_X_:H with a refractive index of 2.05. It is considered that the absorption coefficient in the ultraviolet range increases more with bottom layer thickness, resulting in a lessened passivation effect. Figure 1 shows the current-voltage [*I*-*V*] characteristics measured. The sample with a 20-nm-thick bottom layer has the best performance of 17.8% efficiency. Table 1 shows that the cells with double-layer antireflection coatings have better open circuit voltage and fill factor than those with single-layer antireflection coating. There is hardly any change in the short circuit current, as seen in Table 1. Therefore, the cell efficiency improves when the double stack antireflection coating is used. The solar cell with a 20-nm-thick bottom SiN_X_:H and 60-nm-thick top SiN_X_:H has the longest lifetime and the highest efficiency.

**Table 1:** Solar cell characteristics

	***V***_**oc**_ (mV)	***J***_**sc**_ (mA/cm^2^)	Fill factor (%)	Efficiency (%)
Reference	625	35.7	76.8	17.2
Double stack	626	36.3	78.3	17.8

*V*_oc_, open circuit voltage; *J*_sc_, short circuit current density.

**Figure 2:**
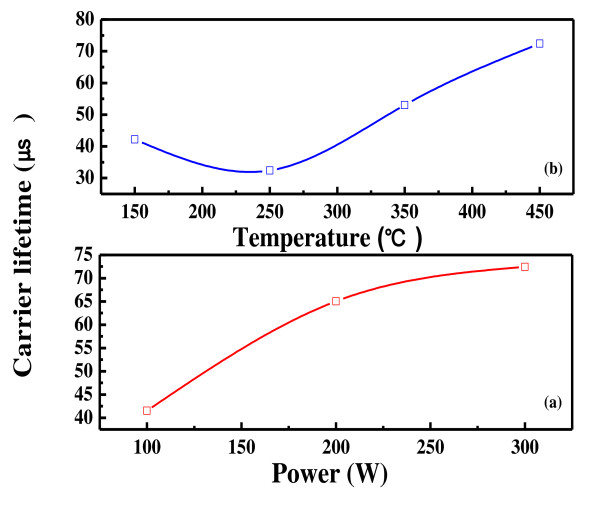
**Carrier lifetime of the SiN_X_:H film at various powers (a) and substrate temperatures (b)**.

The results show that solar cells with SiNx/SiNx double stack antireflection coating have good efficiency. However, it is more important that it fulfills the role of an ARC of a solar cell when exposed to the outside environment, especially for mass-produced solar cells. The endurance dependence on the refractivity and structure of the ARC material is tested. First, the optimized deposition conditions are found by varying the deposition power and temperature and measuring the carrier lifetimes. Figure 2a, b depicts the variation of the carrier lifetime of the SiN_X_:H film deposited on an n-type circle Si wafer, as a function of deposition power (in Watts) and deposition temperature. The deposition temperature is varied from 150°C to 450°C. The carrier life time is low when deposited at a temperature of 150°C to approximately 250°C. It is even lower when deposited at 250°C. However, the carrier lifetime increases when the temperature is above 350°C. Figure 2b demonstrates the effective minority carrier lifetime (*τ*_eff_) of the SiN_X_:H films at various plasma powers while keeping the substrate temperature at 450°C and the gas ratio at 0.88. The plasma poser is changed from 100 W to 300 W. The films deposited using a plasma power of 180 mW/cm^2 ^have the highest value of the effective minority carrier lifetime, around 72 μs. Thus, the preferred substrate temperature and plasma power suitable for SiN_X_:H film deposition for solar cell fabrication are chosen to be 450°C and 180 mW/cm^2^, respectively. For each sample, the refractive index is measured by an ellipsometer (VASE^®^, J.A. Woollam Company, Lincoln, NE, USA; 240 nm <*λ *< 1700 nm).

**Figure 3:**
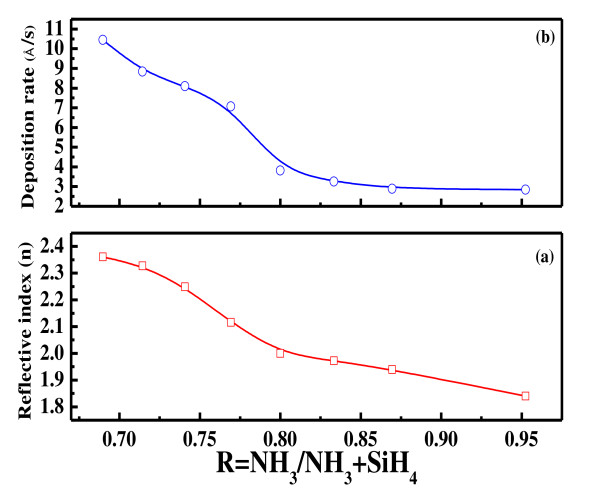
**Refractive index (a) and deposition rate (b) of SiN_X_:H film at various NH_3_/NH_3_+SiH_4 _gas ratios**.

The dependence of refractive index and deposition rate on the gas ratio is an important factor to determine the PECVD deposition conditions. Figure 3a, b depicts the variation of the refractive index (*n*) and deposition rate of the SiN_X_:H film deposited on an n-type circle Si wafer as a function of the NH_3_/NH_3_+SiH_4 _gas ratio. Figure 3a shows that the refractive index of the film decreases from approximately 2.3 to 1.8, with an increase in the NH_3_/NH_3_+SiH_4 _gas ratio from 0.68 to 0.95. From Figure 2b, we can observe a fall in deposition rate of the SiN_X_:H films from 10.45 Å/s to 2.85 Å/s, with an increase in the NH_3_/NH_3_+SiH_4 _gas ratio from 0.68 to 0.95. A low refractive index of 1.84 and a low deposition rate of 2.85 Å/s are obtained for samples with a low silane (SiH_4_) flow rate. When the silane flow rate is increased, Si content in the deposited films is increased, enhancing the refractive index value and the deposition rate. As the gas ratio, *R*, increases, the film thickness decreases since the nitrogen atoms within the thin film are increased as NH_3 _is increased. The N-H bonds increase making the film n-rich, resulting in the decrease of refractive index [10].

**Figure 4:**
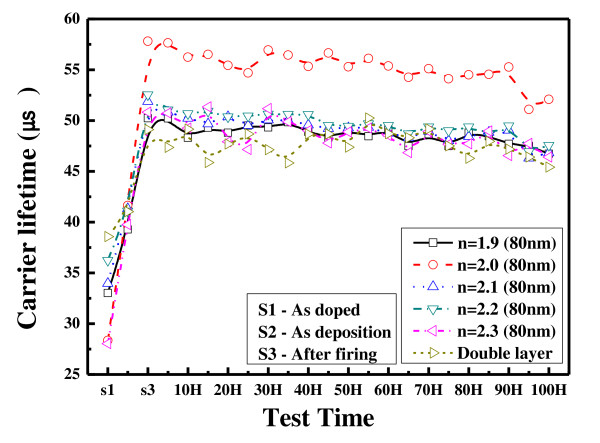
**Measured carrier lifetimes after heat and damp test for 100 h**.

Figure 4 shows the measured carrier life times after the heat and damp test for 100 h. In all cases, the carrier lifetime increases after firing and slowly decreases as the 100-h test is performed. The sample with refractive index of 2.0 has the highest lifetime of 57.8 μs after firing. The effect of passivation of the hydrogenated SiNx increases after firing due to the diffusion of hydrogen into the silicon. The hydrogen bonds with a dangling bond of silicon result in good passivation [11]. The lifetime of the double stack thin film is 49 μs, somewhat low compared to the film with a refractive index of 2.0. However, it is comparable to other thin films, proving that the double stack film has the effect of passivation. It is known that the double stack film has good passivation due to the effect of the passivation of the bottom layer [12]. After the 100-h stress test, the lifetime of the thin film with a refractive index of 2.0 decreased from 57.8 μs to 52 μs, i.e., it decreased by 9.9%. For thin films with refractive indices of 1.9 to approximately 2.3, the average lifetime decay rate is 8.9%. For the double stack film, the life time changes from 49.1 μs to 45.4 μs, showing that it decreases by 7.5%. It is estimated that the double stack film could endure the applied stress since the thin film with a refraction index of 2.3, which serves as a good passivation, can protect the film with a refraction index of 1.9. It is also predicted that an actual solar cell with the double stack film would have better passivation.

**Figure 5:**
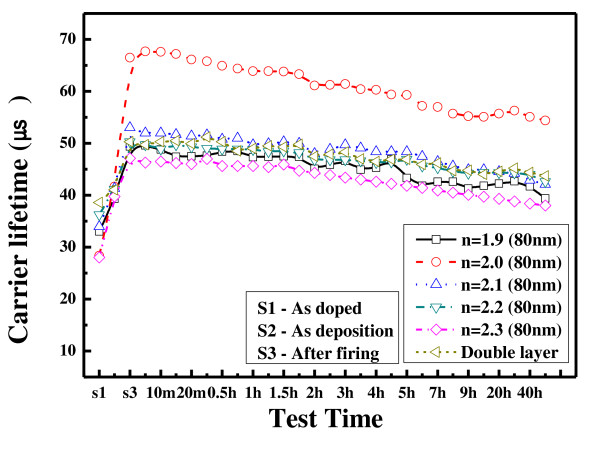
**Measured carrier lifetimes after UV exposure for 50 h**.

Figure 5 shows the measured carrier life time after UV exposure for 50 h. Direct UV exposure for 50 h is similar to 5 months exposure in real life [13]. The sample with a refractive index of 2.0 has the highest lifetime of 66 μs after firing, as in Figure 3.

The double stack film has a lifetime of 50 μs, which is not worse compared with other thin films. The lifetime of the film with a refractive index of 2.0 decreases from 66 μs to 54 μs after 50 h of UV exposure. Its decay rate is 18.2%. The lifetime of the double stack film decreases by 13.1% from 50 μs to 44 μs. For other thin films with refractive indices from 1.9 to approximately 2.3, the average lifetime decay rate is 18.7%, which is higher than the double stack film by more than 5%. This proves that the solar cells with double stack antireflection film are stable against UV light.

**Figure 6:**
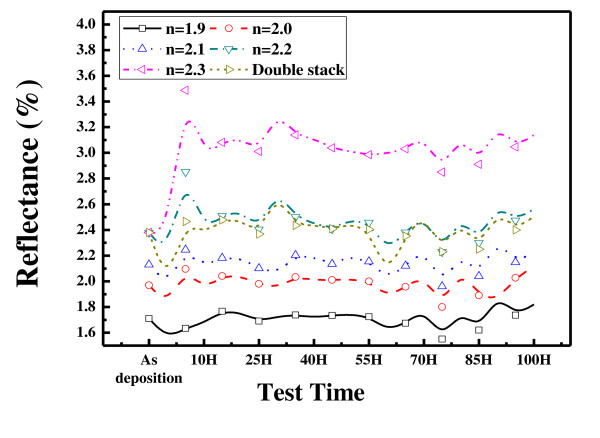
**Reflectance of the SiN_X_:H film after heat and damp test**.

Figure 6 shows the changes of reflectivity after 100 h of heat and damp test. It can be seen that the samples with low refractive index have low reflectivity. The absorption coefficients are proportional to the density of the material. When the refractive index is high, the absorption coefficients are high, the transmittance is low, and it is possible to produce thin films with high reflectivity.

A thin film with a refractive index of 2.3 has a high reflectivity of 3%, and a film with a refractive index of 1.9 has a reflectivity below 2%. In all cases, the reflectivity remains almost unchanged after the heat and damp test. This means that the thin films fabricated by PEVCD are not directly affected by the rapid change in temperature and the humidity.

**Figure 7:**
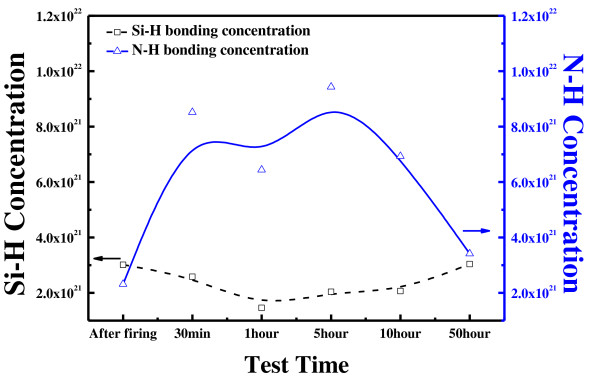
**Hydrogen concentration of the double stack SiN_X_/SiN_X _film after UV exposure test**.

Figure 7 shows the Si-H and Si-N bonding concentration changes with the UV exposure time. Si-H bonding at 2, 170 cm^-1 ^and N-H bonding at 3, 340 cm^-1 ^are indentified using the transmission mode of the FT-IR analyzer [14]. The relative concentrations of Si-H and N-H bonding are calculated according to Beer's rule. The Si-H bonding concentration changed from 3.01 × 10^21 ^cm^-3 ^to 3.04 × 10^21 ^cm^-3^, and the N-H bonding concentration changed from 2.31 × 10^21 ^cm^-3 ^to 3.41 × 10^21 ^cm^-3 ^after UV exposure. There is more change in the N-H bonding concentration. It is assumed that the hydrogen within the thin film is diffused into the silicon during the firing process and the hydrogen bonds with dangling bonds, resulting in good passivation and stability. However, the nitrogen atoms remain within the thin film and get excited during the short periods of UV exposure. However, it is seen that they return to the stable bonding concentration as the UV exposure time is prolonged. Although there is change in the N-H bonding, the Si-H bonding is in a stable state after firing, and the passivation is not affected much, as seen in Figure 5.

## Conclusion

It is known that solar cells with double stack antireflection coating have better efficiency than those with single-layer ARC. The same results are obtained in our experiments. The solar cell with a 60-nm/20-nm SiN_X_:H double stack antireflection coating has 17.8% efficiency, while that with an 80-nm SiN_X_:H single-layer antireflection coating has 17.2% efficiency. The improvement of the efficiency is due to the effect of better passivation and better antireflection of the double stack antireflection coating. However, studies on the stability against outside environment for double stack ARC are seldom conducted.

The effects of temperature, humidity, and UV exposure on the SiN_X_:H thin films with different gas ratios were investigated, and the stability of the double stack antireflection coating thin film was examined. First, single-layer antireflection coatings were studied to establish the deposition conditions, and the results were applied to the double stack antireflection coating. The passivation of the thin films with various refractive indices was also studied. After the temperature and humidity test for 100 h, the carrier lifetime of the thin film decreased by 7.5%. The lifetime decreased by 13.1% after the UV exposure test. These are better results than those obtained for the average of single layers, 8.9% after the heat and damp test and 18.72% after UV exposure. The stability of double stack antireflection coatings has been experimentally confirmed.

## Competing interests

The authors declare that they have no competing interests.

## Authors' contributions

YL proposed the original idea, carried out the synthesis and analysis of the experiment, and wrote the first draft of the manuscript. DG carried out most of the experiments with YL and shared his idea with the other authors. NB and YJL detailed the original idea and modified the first draft of the manuscript. JY designed and coordinated the whole work and finalized the manuscript. All authors read and approved the final manuscript.
